# Development of a Novel Anesthesia Airway Management Robot

**DOI:** 10.3390/s21238144

**Published:** 2021-12-06

**Authors:** Xuesong Ma, Bo Pan, Tao Song, Yanwen Sun, Yili Fu

**Affiliations:** 1 School of Life Science and Technology, Harbin Institute of Technology, Harbin 150001, China; 120001712@hrbmu.edu.cn; 2 The Fourth Clinical Medical School, Harbin Medical University, Harbin 150001, China; 3 State Key Laboratory of Robotics and Systems, Harbin Institute of Technology, Harbin 150001, China; mepanbo@hit.edu.cn (B.P.); 16b908032@stu.hit.edu.cn (T.S.); syw.hit@hit.edu.cn (Y.S.)

**Keywords:** positive pressure ventilation, general anesthesia, medical robot

## Abstract

Non-invasive positive pressure ventilation has attracted increasing attention for air management in general anesthesia. This work proposes a novel robot equipped with two snake arms and a mask-fastening mechanism to facilitate trachea airway management for anesthesia as well as deep sedation and to improve surgical outcomes. The two snake arms with supporting terminals have been designed to lift a patient’s jaw with design optimization, and the mask-fastening mechanism has been utilized to fasten the mask onto a patient’s face. The control unit has been developed to implement lifting and fastening force control with safety and robustness. Loading experiments on the snake arm and tension experiments on the mask-fastening mechanism have been performed to investigate and validate the performances of the proposed anesthesia airway management robot. Experiments on a mock person have also been employed to further verify the effectiveness and reliability of the developed robot system. As an early study of an anesthesia airway management robot, it was verified as a valid attempt to perform mask non-invasive positive pressure ventilation technology by taking advantage of a robotic system.

## 1. Introduction

At present, the establishment of an artificial airway is necessary for ventilation in patients under general anesthesia [1]. Inserting a ventilator duct into the patient’s trachea to establish an “artificial airway” is often used in general anesthesia operations. This is known as intermittent positive pressure ventilation (IPPV). IPPV could cause airway and pharyngeal compression injuries, and increase medical risks [2]. In contrast, non-invasive positive pressure ventilation (NPPV) can provide a reduced number of complications related to mechanical ventilation compared with IPPV [3,4]. NPPV is usually applied to awake patients, because patients are prone to experience pharyngeal airway obstruction after losing consciousness [5]. If the jaw is raised into an appropriate posture by hands, the patient’s airway can be kept unobstructed. In this condition, NPPV can be applied to general anesthesia surgeries. However, satiated patients who are at risk of gastric contents reflux are not suitable for these surgeries as the mask cannot isolate the trachea. Additionally, the air pressure should be controlled in a suitable range to avoid gastric distension [6]. Air leakage through the mouth is the leading cause of NPPV failure [7,8]. Thus, NPPV is only used as a temporary ventilation method during the induction of general anesthesia [9,10]. In recent years, clinical studies on NPPV have attracted increasing attention, especially in regard to the usage of robot technology.

The application of medical robots in the field of anesthesia has been extensively reported [11,12]. The research directions mainly focus on the autonomous control of anesthesia depth [13,14]. Considering the advantage of NPPV, some studies have attempted to mechanically support the chin during general anesthesia. With the development of technology, using devices instead of hands to open the airway is becoming a trend in the research [15,16]. In some studies, a jaw support device was developed. With the Jaw Elevation Device (JED), airway patency can be maintained when patients are under general anesthesia or deep sedation. It is a great step forward in improving the safety of spontaneous breathing in patients. Additionally, the technique greatly expands the application scope of spontaneous respiratory in the vein of general anesthesia surgeries [17,18]. However, this medical device is typically rigid in structure and inconvenient to adjust. Meanwhile, the above-mentioned devices suffer from a lack of mask-fastening functions; thus, they cannot be applied directly in NPPV during general anesthesia.

To address these challenges, a robot-assisted NPPV system has been proposed and developed. The robotic system is equipped with a couple of snake arms and a mask-fastening mechanism. Patients’ upper airways are opened by the robot, and the air is fluently transmitted by the mask. In clinical practice, patients take a supine position. The anesthesiologist can operate jaw lifts by using the tentacles of the snake arm, with appropriate force, to considerably adjust the patient’s head posture. After the patient’s head posture is determined, the snake arms would be tightened to keep the patient’s airway unobstructed, and then the mask would be fastened tightly according to the fastening mechanism. Subsequently, NPPV in general anesthesia can be conducted with the assistance of the robot. The proposed robot system overcomes the shortcomings associated with the existing technology. This design can conveniently and safely replace the manual implementation of mandibular airway management by an anesthesiologist, making the operational procedure more effective.

## 2. Materials and Methods

### 2.1. The Clinical Needs for an Anesthesia Airway Management Robot

The robot should have functions equivalent to manual NPPV airway management. Additionally, it should be suitable for patients who have no risk of vomiting and aspiration in short general anesthesia operations. During the procedure, the mandible should be lifted to present the anatomical position of the mandibular reverse occlusion, so as to relieve the patient’s glossocoma after general anesthesia and ensure the airway is unobstructed. Meanwhile, the mask should be fastened tightly to the patient’s face to transmit air smoothly. If any risks occur during an operation, traditional IPPV methods can be used immediately as a remedy.

According to the above-presented clinical scenarios, the design of an anesthetic airway management robot should meet the following four requirements to achieve the auxiliary NPPV procedure:

1. Requirements for support strength. The designed manipulator should possess a convenient and robust locking function and provide sufficient support to ensure airway management motions. The joint locking strength of the manipulator should be able to resist the pressure of at least 30 N.
2. Degree of manipulator freedom requirement. The design of the manipulator should provide a universal support function with sufficient flexibility.
3. Requirement for mask fastening. The pressure provided by the mechanical structure to fasten the mask could be larger than 15 N.
4. Safety requirements. The manipulator should also provide a convenient and reliable manual unlocking function to prevent unexpected mechanical failures.

### 2.2. Structure Design of Anesthesia Airway Management Robot

**Figure 1:**
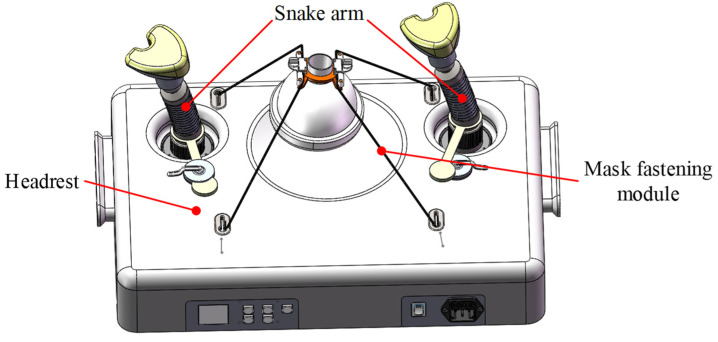
An overview of the proposed anesthetic airway management robot.

The anesthesia airway management robot has been designed to meet the design requirements mentioned above, as illustrated in Figure 1. The designed robot mainly comprises a pair of snake arms with a universal support function, a mask-fastening module, and a headrest. Two snake arms are vertically installed on the baseplate of the headrest. A tentacle that lifts the jaw is set at the arm end to simulate the hand motions used to maintain airway management. Thus, the patient’s mandible can be supported, and the patient’s airway can maintain an opening state during the operation. The mask-fastening module is composed of a wire rope drive system and a mask. The motor inside the headrest controls the tightness of the wire rope, and thus the pressure applied to the patient’s face can be adjusted. The headrest supports the patient’s neck and head and helps patients in a supine position from a head back elevation posture. This headrest is equipped with both electric and manual unlocking functions of the snake arms to ensure the safety and reliability of the proposed system.

**Figure 2:**
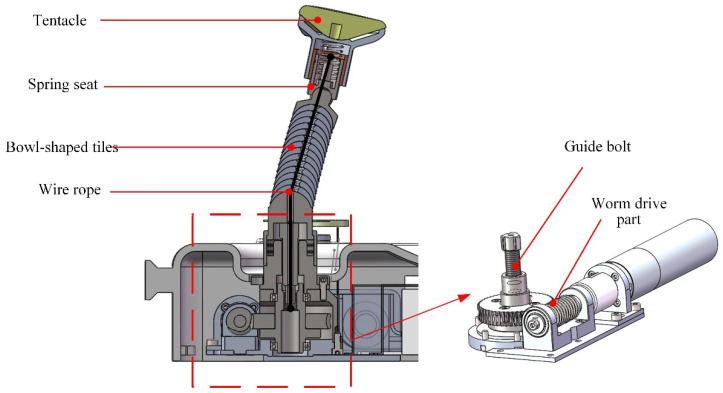
The structural design details of the proposed snake arm.

The continuum snake arm consists of a tentacle, a spring seat, bowl-shaped tiles, a wire rope, and a guide bolt, as illustrated in Figure 2. The universal support structure of the snake arm is the core part of the robotic system. It is designed with multiple joints to form the continuum configuration and is composed of several bowl-shaped tiles in a serial connection. A through hole has been made in the middle part of each bowl-shaped tile, enabling the driving wire rope to carry. The wire rope runs through each joint of the whole arm, and its tail has been fixed at the worm wheel stretching mechanism designed at the system base. When the worm gear is operated, the wire rope can be tightened, and the whole joint of the manipulator can be tightened and locked. On the contrary, when the wire rope is relaxed, the locking state of each joint of the snake arm will be released. The tentacle at the distal end of the snake arm is provided with a specific surface structure corresponding to the mandible contact part to ensure uniform pressure and protect the patient’s mandible. The spring seat that connects with the tentacle provides the elastic pressure and realizes elastic force application within a specific range.

**Figure 3:**
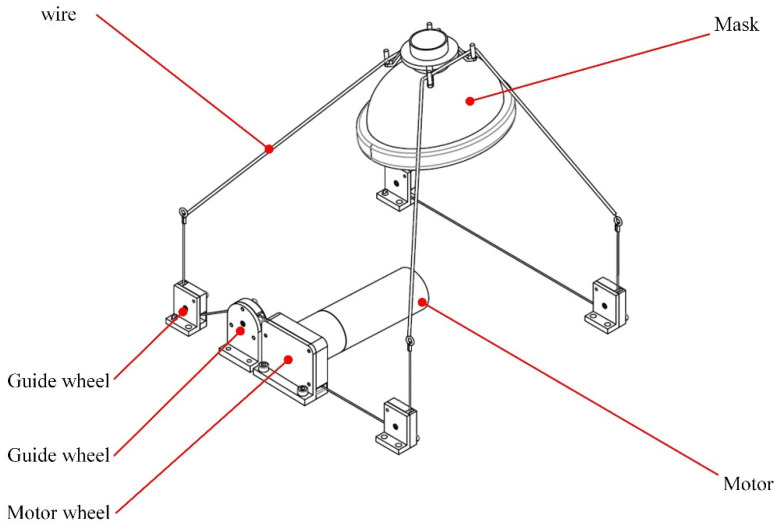
The structural profile of the proposed mask-fastening module.

The mask-fastening module comprises a motor, a mask, 6 wire wheels, and several steel wire ropes, as illustrated in Figure 3. Two wires run through wheels to press the mask, and their tails have been fixed to the motor. Wires are tightened when the motor rotates; thus, pressure on the mask can be increased. If wires are released, the motor will power off.

With the designed system, the operating surgeon can manually adjust the snake arms and apply forces symmetrically to the bilateral mandibular of the patient through the distal tentacles. Subsequently, the driving motor can tighten the wire rope and fasten the snake arms to meet the airway management requirements. The mask will be pressed to fit the patient’s face, and the motor will rotate to tighten the wires to form suitable tension. The proposed robotic system can replace the manual action of airway management through the above modules.

### 2.3. Design Optimization of the Bowl-Shaped Tiles

**Figure 4:**
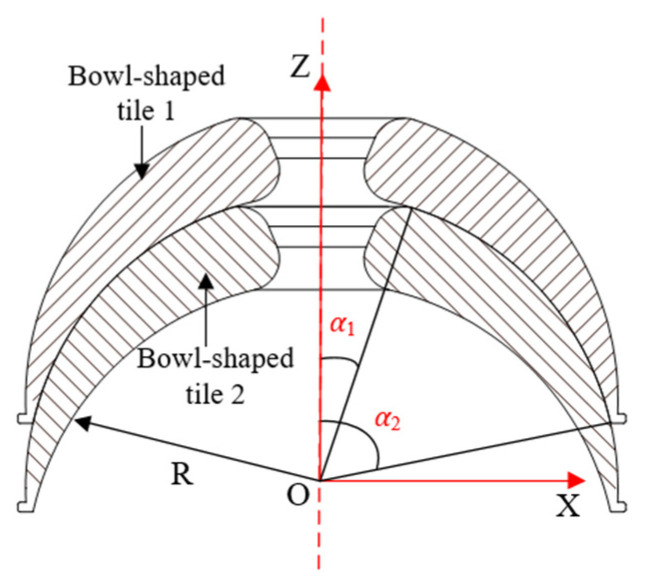
Illustration of the contact between two bowl-shaped tiles.

The locking function of the snake arm is realized by controlling the motor to drive the worm part to tighten the wire rope. According to the operational characteristics of the snake arm, the tension required by the wire rope is closely related to the friction between the bowl-shaped tiles. Therefore, optimization of the bowl-shaped tiles can play an essential role in structure design. The frictional contact between two bowl-shaped tiles has been illustrated in Figure 4.

Referring to the calculation method of spherical friction torque [19], the friction torque of a bowl-shaped tile rotating around the *z*-axis is:

(1) $$\begin{array}{l} M_{Z}=\int \mu \sigma_{\alpha }dsR\sin\alpha \\ \;\;=\int_{\alpha_{2}}^{\alpha_{1}}\mu \frac{3F}{2\pi R^{2}\left(\cos^{3}\alpha_{1}-\cos^{3}\alpha_{2}\right)}\cos\alpha R\sin\alpha ds\\ \;\;=\int_{\alpha_{2}}^{\alpha_{1}}\mu \frac{3FR}{\left(\cos^{3}\alpha_{1}-\cos^{3}\alpha_{2}\right)}\cos\alpha \sin^{2}\alpha d\alpha \\ \;\;=\frac{\mu FR}{\cos^{3}\alpha_{1}-\cos^{3}\alpha_{2}}\left(\sin^{3}\alpha_{2}-\sin^{3}\alpha_{1}\right) \end{array}$$

where *R* denotes the radius of the friction sphere, *μ* denotes the friction coefficient between bowl-shaped tiles, and *F* expresses the axial load. *α*_1_ represents the angle between the connection line from the center of the ball to the upper edge of bowl-shaped tile 1 and the *z*-axis, and *α*_2_ is the angle between the connection line from the center of the ball to the lower edge of bowl-shaped tile 1 and the *z*-axis.

Since *x* and *y* are interchangeable, the rotation torques of the tiles around the *x*-axis and *y*-axis are equal, that is, *M_x_* = *M_y_*. The friction torque of the bowl-shaped tile around the *x*-axis is:

(2) $$\begin{array}{l} M_{x}=\int_{\alpha_{1}}^{\alpha_{2}}\int_{0}^{l_{a}}\int_{n_{0}}^{R}4\mu \sigma_{s}dndlRd\alpha \\ \;\;=\int_{\alpha_{1}}^{\alpha_{2}}4\mu \frac{3F}{2\pi R^{2}\left(\cos^{3}\alpha_{1}-\cos^{3}\alpha_{2}\right)}\cos\alpha \left(R-R\cos\alpha \right)\frac{\pi R\sin\alpha }{2}Rd\alpha \\ \;\;=\int_{\alpha_{1}}^{\alpha_{2}}\frac{3\mu F}{\left(\cos^{3}\alpha_{1}-\cos^{3}\alpha_{2}\right)}\cos\alpha \sin\alpha (1-\cos\alpha )Rd\alpha \\ \;\;=\frac{3\mu FR\left(\sin^{2}\alpha_{2}-\sin^{2}\alpha_{1}\right)}{2\left(\cos^{3}\alpha_{1}-\cos^{3}\alpha_{2}\right)}-\mu FR \end{array}$$

According to the design requirements, 15° < *α*_1_ < 40°, 60° < *α*_2_ < 80°, it is obtained that *M_z_* is smaller than *M_x_* after theoretical analysis. Besides, it can be found that the forms of the equations of *M_z_* and *M_x_* are very similar, which means that they also share a similar change tendency. Thus, considering the convenience of simulation analysis, it is better to choose the smaller one (*M_z_*) as the working condition of simulation.

**Figure 5:**
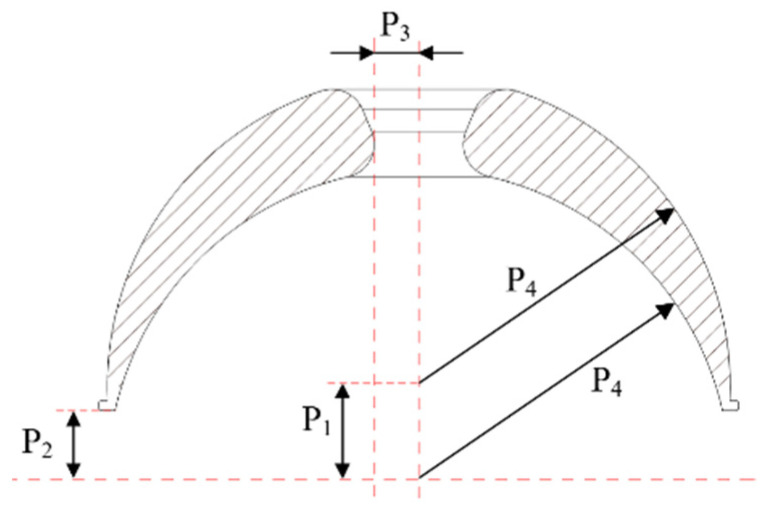
Illustration of structural parameters to be optimized in a bowl-shaped tile.

As shown in Equations (1) and (2), the friction torque of bowl-shaped tiles is related to *R*, *μ*, *α*_1_, and *α*_2_. Because *R* is related to *α*_1_ and *α*_2_, it is hard to optimize with these parameters. Therefore, the inner and outer circle center distance, P_1_, *z* tangential distance, P_2_, center hole radius, P_3_, and inner and outer circle radii, P_4_, are selected as the structural parameters to be optimized, as illustrated in Figure 5.

The variation range of structural parameters is shown in Table 1.

**Figure 6:**
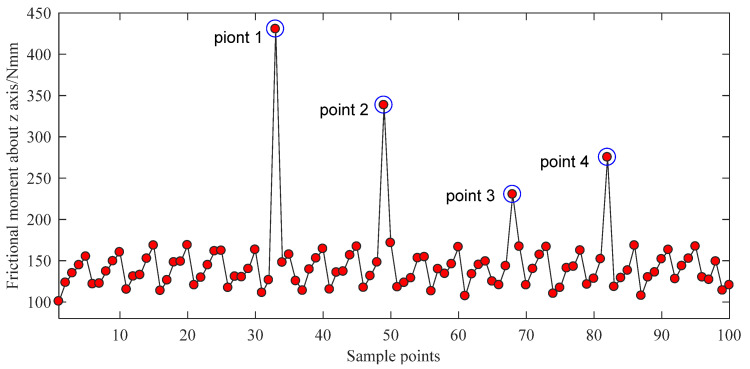
Bowl-shaped tiles’ friction torques of 100 sample points.

**Figure 7:**
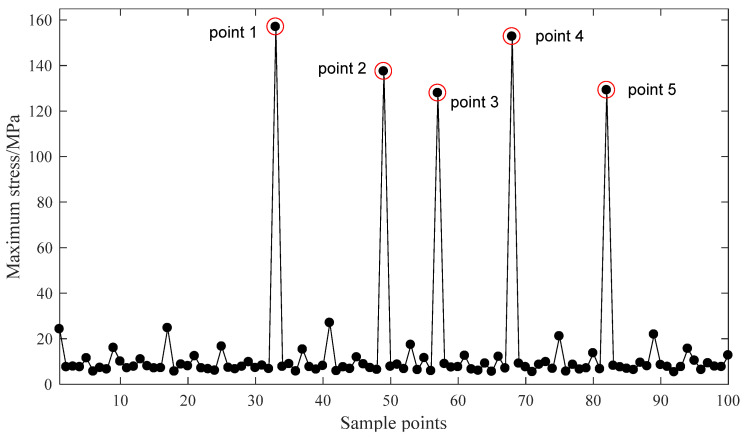
Bowl-shaped tiles’ maximum stress of 100 sample points.

For the material of the bowl-shaped tiles 17-4PH is chosen, and 100 N of axial force is applied on the surface of bowl-shaped tiles. The design optimization process is conducted using ANSYS Workbench 17.0 (ANSYS Inc., Pittsburgh, PA, USA). The direct optimization method has been utilized and requires an appropriate sample size to make the calculation accurate. Therefore, the best adaptive tetrahedral mesh division approach is adopted. The element size on the top of the bowl-shaped tiles is set as 1.5 mm, and the element size in the bottom of the bowl-shaped tiles is set as 1 mm. The maximum friction torque is selected as the main optimization objective. The screening method is selected as the optimization algorithm. The equivalent stress of the material is selected as the constrained condition. According to the variation range of optimized parameters, 100 groups of data are selected as samples through ANSYS software. The optimization results are shown in Figure 6 and Figure 7.

As shown in Figure 6 and Figure 7, there are five singular points for the optimization analysis. Models were built using the parameters of these five singular points. It is found that the extreme values are caused by two main factors. First, some models have extremely thin edges. Second, some singular points of the grid are found on the other models. Therefore, the data of these five points should be abandoned. By reordering the rest results, the optimal parameters P_1_ = 4.1551, P_2_ = 3.1031, P_3_ = 2.0355, and P_4_ = 13.836 can be easily found. After rounding this dataset, the optimal structural parameters of the bowl-shaped tiles are obtained, as shown in Table 2.

### 2.4. Control System Design of the Anesthesia Airway Management Robot

**Figure 8:**
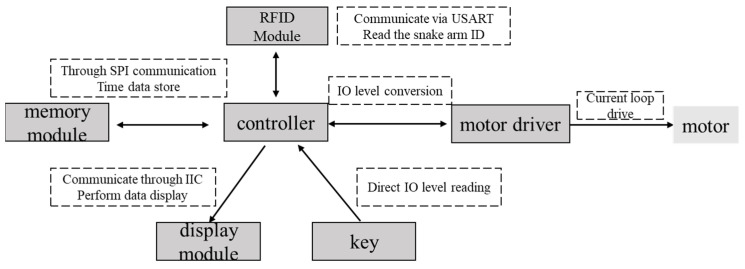
The presented configuration of the control system.

According to the requirements of the anesthesia airway management robot, the control scheme is designed with the following modules, as shown in Figure 8.

**Figure 9:**
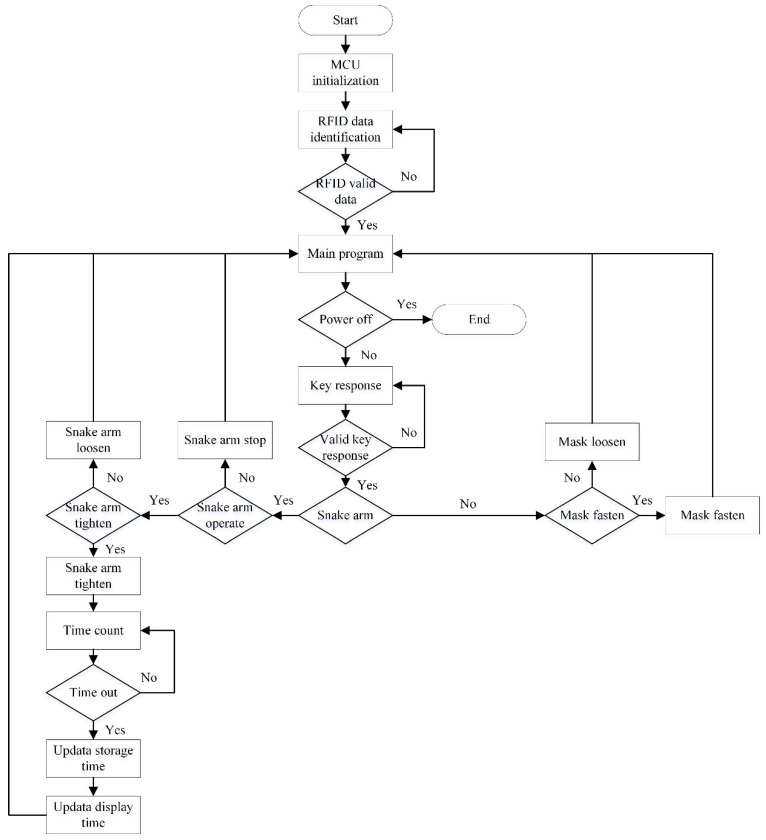
The presented software flow of the control system.

The control system consists of a controller (STM32G031), three motor drivers, an RFID module, a display module, a memory module, and user input keys. The controller receives information from the input keys and converts the information to a group of high- and low-level output voltages. The motor drivers receive the information from the controller and perform operations to control the snake arms and mask-fastening module. The RFID module is mounted on the snake arms and contains their identity information. With the wireless communication part, the RFID module transfers the snake arm ID to the controller. The display module is used to display motor torque information and the usage time of the snake arms. The software workflow to realize control functions has been illustrated in Figure 9.

## 3. Results

The proposed anesthetic airway management robot was tested and validated to investigate its technical and clinical performance. Loading experiments on the snake arm and tension experiments on the mask-fastening mechanism were performed. Simulation experiments on a mock person have also been employed to further verify the effectiveness and reliability of the developed robot system.

### 3.1. Loading Experiment of the Snake Arms

In clinical practice, doctors usually decide the best lifting force according to the status of airway patency and the pressure intensity of fingers on the lifting part. According to design requirements, the mandibular lifting force should be 30 N as the reference data of the lifting force when the patient’s jaw is lifted by an anesthesia airway management robot alone.

**Figure 10:**
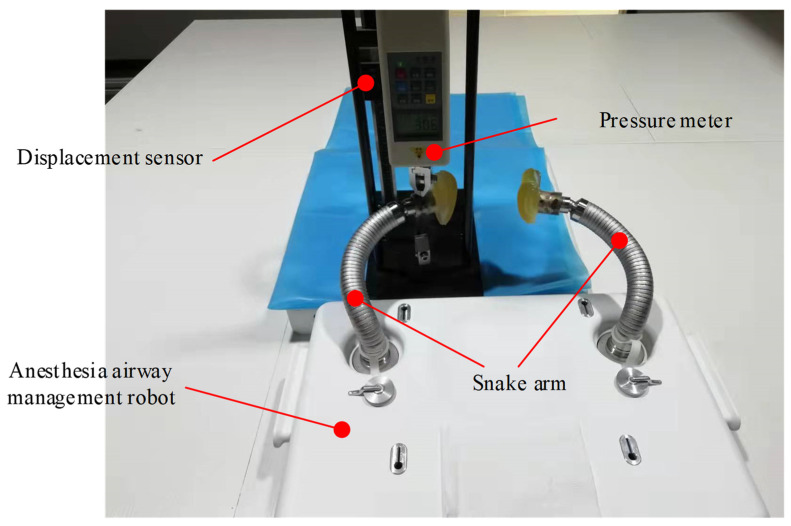
The platform for the loading experiment of the snake arms.

A test platform for the loading experiments of the snake arms was designed. It mainly consists of the designed anesthesia airway management robot, a displacement sensor, and a pressure meter, as shown in Figure 10. A distal force loading of 30 N was applied to the upper end of the snake arms six times, and both the initial position and final position of the distal end of the snake arms were recorded, as detailed in Table 3. The mean displacement, standard deviation, and maximum displacement values from the six-group experiments were calculated, as shown in Table 4. When the snake arms support a load of 30 N, the distal displacement was less than 5 mm, meeting the clinical requirements.

### 3.2. Tension Test of the Mask-Fastening Module

**Figure 11:**
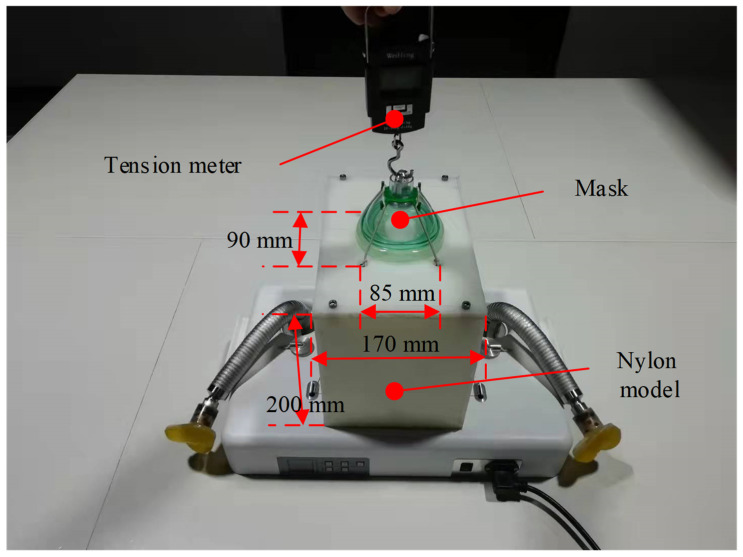
The platform for the tension test of the mask-fastening module.

The design requirements of the mask-fastening module are as follows: When the oxygen mask is pulled upward in a vertical direction, the tension of the mask should be larger than 15 N if it leaves the contact surface. A platform for the tension test of the mask-fastening module was designed, as shown in Figure 11. A nylon model with a width of 170 mm and a height of 200 mm was made to simulate the size of a human head. This model has been fabricated with four holes on the surface to pass the wire. The distance between the two holes in the front row was 85 mm, and the distance along the other direction was 90 mm. The elastic wires were connected to wire ropes through four holes to pull the mask. For each experiment, wires were pulled to the lowest position, and the mask was pulled through the tension meter to move the mask away from the contact surface. Meanwhile, the tension was measured when the mask was away from the contact surface.

Ten experimental trials have been conducted on the mask to obtain the tension force values. The collected force results have been presented in Table 5. Further analysis has been performed to determine the mean value, variance, and maximum as well as minimum values, as summarized in Table 6. The stable tension force that requires separating the mask from the contact surface was around 22.594 N, which met the design requirements of the tension force.

### 3.3. Simulation Study of the Anesthesia Airway Management Robot

**Figure 12:**
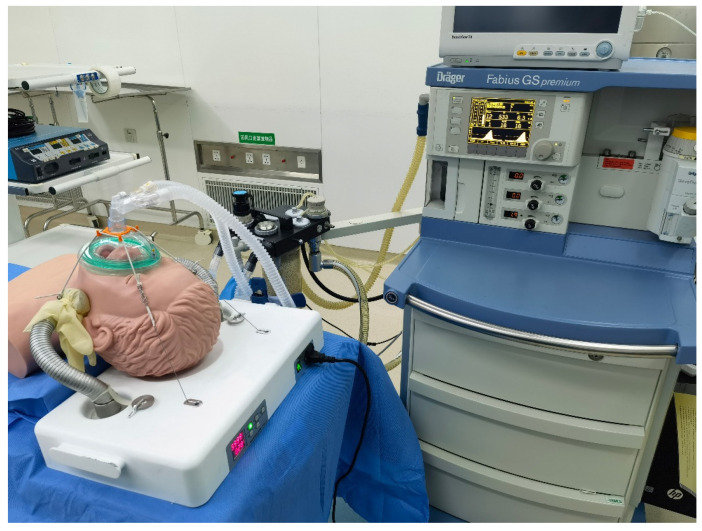
Simulation experiments of anesthesia airway management robot.

The simulation experiments were conducted on a mock person in the Fourth Affiliated Hospital of Harbin Medical University. The experimental setup mainly included the designed anesthesia airway management robot, one operation table, one multi-functional anesthesia machine, and a mock person used for CPR. The mock person was supine relative to the anesthesia airway management robot, with his head in a backward position. A group of mechanical ventilation clinical simulated experiments was performed, and the effectiveness of the new airway management method in the mock person application was evaluated. The snake arms were initially configured with a relaxed state. The anesthesiologist controlled both sides of the snake arm tentacles to lift the mock person’s mandibular (between the mandibular angle and earlobe). When the posture and position were significantly confirmed, the snake arms would be locked. Subsequently, the mask would be pulled and fastened on the mock person’s face to complete the mask airway management operations with assistance from the designed robot. After that, the mock person was ventilated using an anesthesia machine, as shown in Figure 12.

Three tests under the ventilation were performed. The related parameters in terms of the set tidal volume (VTi), the tidal volume under airway management (VTE), respiratory rate (RR), airway peak pressure (PEAK), and ventilation time (T) were collected every 2 min. The above ventilation-related parameters varied in the normal range when the airway was unobstructed, and the mask was well-sealed. The test results of the ventilation-simulated experiment have been summarized in Table 7. In all tests, the VTE values were greater than VTi and the PEAK values were less than 20 cm H_2_O. These experiments were considered successful.

## 4. Conclusions

A novel anesthesia airway management robot that mainly consists of two continuum snake arms, a mask-fastening module, and a headrest has been developed for airway management during general anesthesia. The system can simulate the skills and techniques of anesthesiologists to lift the patient’s jaw and fasten masks during clinical operations to achieve non-traumatic anesthesia. Both the loading experiments and simulation experiments on a mock person have been performed for performance validation. The distal deformation of the continuum snake arms was 0.16 mm under a load of 30 N, and the force to pull the mask away from the contact surface was 22.594 N. During the simulation tests, both the VTE values and PEAK values demonstrated desirable results. As the early phase of the robot development, the proposed anesthetic airway management robot was verified to be valid. In future studies, the comfort and safety of the robot will be further optimized, and the scope of its clinical application will be further investigated [20,21,22].

## Figures and Tables

**Table 1:** Range of structural parameters to be optimized in a bowl-shaped tile.

No.	Parameter	Range/mm
1	Center distance of circle, P_1_	[3.6, 4.8]
2	Tangential distance in z direction, P_2_	[2, 4]
3	Center hole radius, P_3_	[1.5, 2.5]
4	Radii of inner and outer circle, P_4_	[8, 14]

Note: There is a functional relationship between P_1_ and P_2_, P_2_ = P_1_/2. P_3_ is mainly limited according to the diameter of the wire rope. P_4_ is limited by the palm of the medical staff.

**Table 2:** Optimal structural parameters.

No.	Parameter	Initial Value/mm	Optimization Value/mm
1	P_1_	4	4.2
2	P_2_	2.5	3.1
3	P_3_	2.0	2.0
4	P_4_	10	14

**Table 3:** The displacement of the snake arms in the direction of load.

No.	Initial Position (mm)	Final Position (mm)	Displacement (mm)
1	12.48	12.56	0.08
2	25.37	25.65	0.28
3	37.64	37.68	0.04
4	23.61	23.95	0.34
5	28.01	28.13	0.12
6	31.94	32.03	0.09

**Table 4:** Statistical analysis of the load capacity of the snake arms.

Classification	Mean Displacement (mm)	Standard Deviation (mm)	Max Displacement (mm)
Target data	<5	<1	<5
Actual data	0.16	0.11	0.34

**Table 5:** Tensions to separate mask from the contact surface.

No.	Tension (N)	No.	Tension (N)
1	23.079	6	23.422
2	23.520	7	21.903
3	21.903	8	23.128
4	22.344	9	22.589
5	21.805	10	22.246

**Table 6:** Statistical analysis of mask-fastening tensions.

Classification	Mean Tension (N)	Maximal Tension (N)	Minimum Tension (N)
Target data	20–30	<30	>20
Actual data	22.594	23.52	21.805

**Table 7:** Test results of the ventilation-simulated experiment.

No.	VTi (mL)	RR (breaths/min)	T (min)	VTE x ± s (mL)	PEAK x ± s (cm H_2_O)
1	450	12	10	470.8 ± 7.9	13.8 ± 0.8
2	500	12	10	525.6 ± 9.7	15.6 ± 0.9
3	550	12	10	568.4 ± 8.4	16.6 ± 0.5

## Data Availability

The data supporting reported results can be obtained in this article.
